# Dr. Thomas Nuttall

**Published:** 1860-01

**Authors:** 


The death of DR. THOMAS NUTTALL, so well known by his Genera of North American Plants and the Birds of the United States, took place at his residence, Nutgrove, St. Helena, Lancashire, on the tenth of September. A native of Yorkshire, he was raised as a printer, and emigrated to this country toward the close of the last century. He had attained the age of seventy-three years.

